# 1-(Piperidin-1-yl)-9,10-anthraquinone

**DOI:** 10.1107/S1600536812037713

**Published:** 2012-09-08

**Authors:** Elżbieta Wnuk, Paweł Niedziałkowski, Damian Trzybiński, Tadeusz Ossowski

**Affiliations:** a Faculty of Chemistry, University of Gdańsk, J. Sobieskiego 18, 80-952 Gdańsk, Poland

## Abstract

In the title compound, C_19_H_17_NO_2_, the piperidine ring adopts a chair conformation. The mean planes of the piperidine ring and the anthracene ring system are inclined at a dihedral angle of 38.7 (1)°. In the crystal, adjacent mol­ecules are linked through C—H⋯π and π–π [centroid–centroid distance = 3.782 (1) Å] inter­actions, forming a layer parallel to the *bc* plane.

## Related literature

For general background to and applications of anthraquinone derivatives, see: Alves *et al.* (2004 ▶); Czupryniak *et al.* (2012 ▶); Wang *et al.* (2011 ▶); Yeh & Wang (2006 ▶). For related structures, see: Niedziałkowski *et al.* (2011 ▶); Yatsenko *et al.* (2000 ▶).
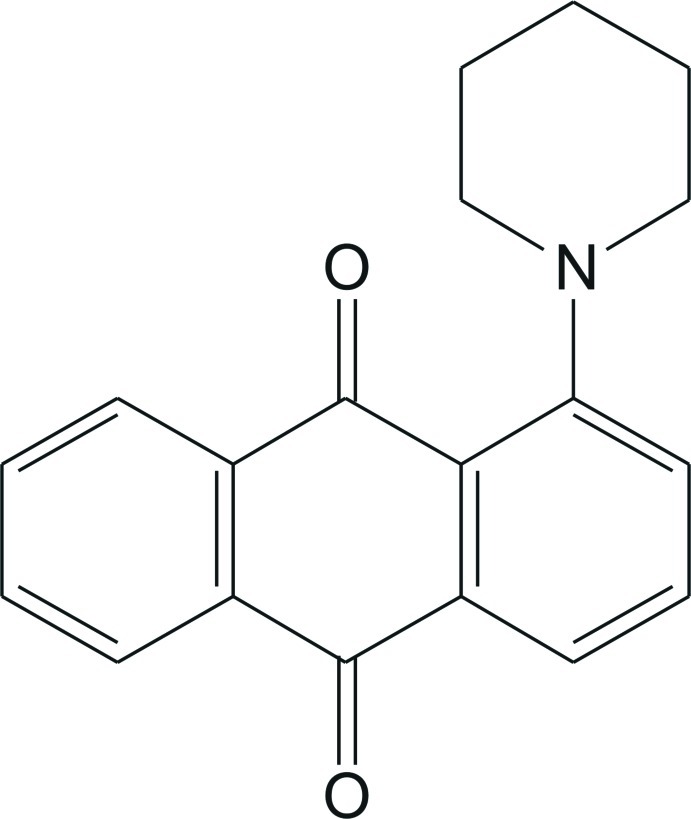


## Experimental

### 

#### Crystal data

C_19_H_17_NO_2_
*M* *_r_* = 291.34
Monoclinic, 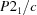

*a* = 16.7798 (4) Å
*b* = 6.84599 (14) Å
*c* = 12.6126 (3) Å
β = 90.723 (2)°
*V* = 1448.75 (6) Å^3^
*Z* = 4
Mo *K*α radiation
μ = 0.09 mm^−1^
*T* = 295 K
0.42 × 0.35 × 0.05 mm

#### Data collection

Oxford Diffraction GEMINI R ULTRA Ruby CCD diffractometer
Absorption correction: multi-scan (*CrysAlis RED*; Oxford Diffraction, 2008 ▶) *T*_min_ = 0.969, *T*_max_ = 0.996
18914 measured reflections
2565 independent reflections
2189 reflections with *I* > 2σ(*I*)
*R*_int_ = 0.023

#### Refinement

*R*[*F* ^2^ > 2σ(*F* ^2^)] = 0.039
*wR*(*F* ^2^) = 0.106
*S* = 1.04
2565 reflections
199 parameters
H-atom parameters constrained
Δρ_max_ = 0.12 e Å^−3^
Δρ_min_ = −0.21 e Å^−3^

### 

Data collection: *CrysAlis CCD* (Oxford Diffraction, 2008 ▶); cell refinement: *CrysAlis CCD*; data reduction: *CrysAlis RED* (Oxford Diffraction, 2008 ▶); program(s) used to solve structure: *SHELXS97* (Sheldrick, 2008 ▶); program(s) used to refine structure: *SHELXL97* (Sheldrick, 2008 ▶); molecular graphics: *ORTEP-3* (Farrugia, 1997 ▶); software used to prepare material for publication: *SHELXL97* and *PLATON* (Spek, 2009 ▶).

## Supplementary Material

Crystal structure: contains datablock(s) global, I. DOI: 10.1107/S1600536812037713/is5185sup1.cif

Structure factors: contains datablock(s) I. DOI: 10.1107/S1600536812037713/is5185Isup2.hkl

Supplementary material file. DOI: 10.1107/S1600536812037713/is5185Isup3.cml

Additional supplementary materials:  crystallographic information; 3D view; checkCIF report

## Figures and Tables

**Table 1:** Hydrogen-bond geometry (Å, °) *Cg*3 is the centroid of the C5–C8/C13/C14 ring.

*D*—H⋯*A*	*D*—H	H⋯*A*	*D*⋯*A*	*D*—H⋯*A*
C2—H2⋯*Cg*3^i^	0.93	2.88	3.685 (2)	146

Symmetry code: (i) 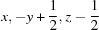.

**Fig. 1.**
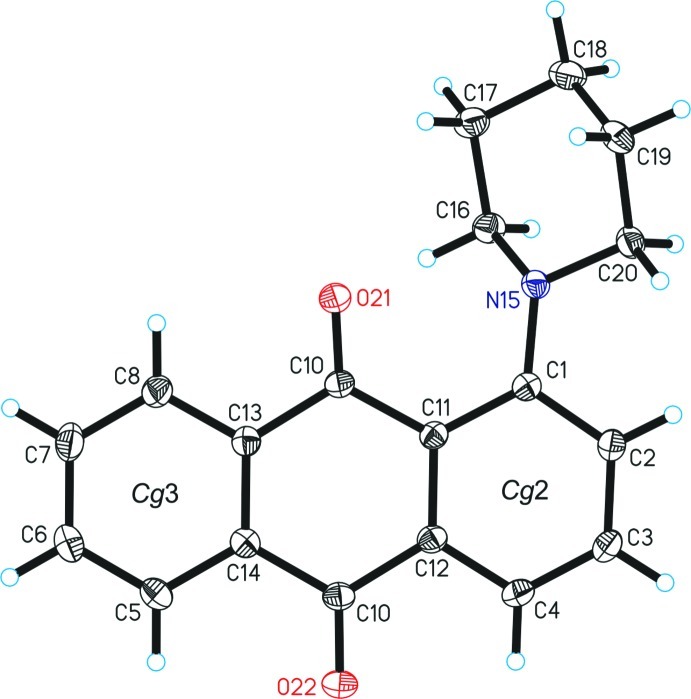
The molecular structure of the title compound showing the atom-labeling scheme. Displacement ellipsoids are drawn at the 25% probability level, and H atoms are shown as small spheres of arbitrary radius. Cg2 and Cg3 are the centroids of the C1–C4/C11/C12 and C5–C8/C13/C14 rings respectively.

**Fig. 2.**
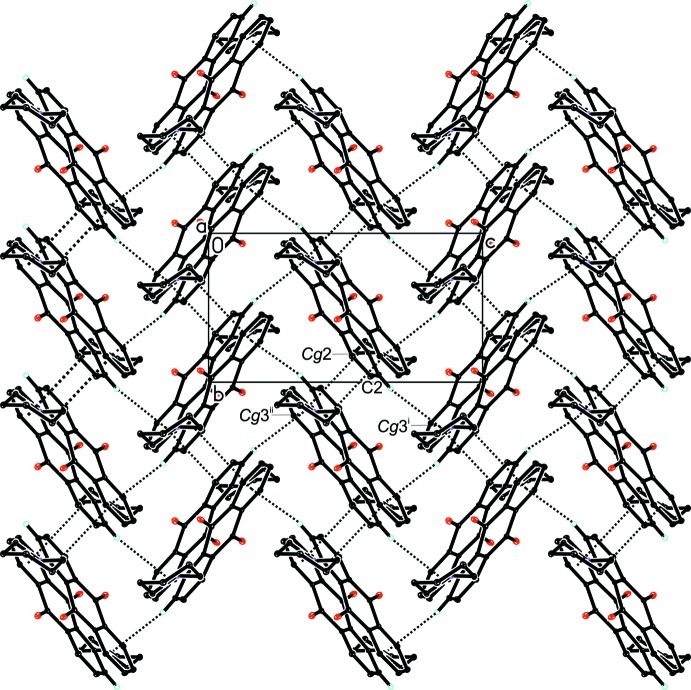
The arrangement of the molecules in the crystal structure. The C—H···π and π–π interactions are represented by dotted lines. H atoms not involved in interactions have been omitted. [Symmetry codes: (i) x, –y + 3/2, z + 1/2; (ii) x, y + 1, z.]

## supplementary crystallographic information

### Comment

Anthraquinones are the most important group of naturally occurring quinones. Both natural and synthetic derivatives of this group of compounds show a wide variety of applications. The color of anthraquinone-based compounds is partially associated with the anthraquinone nucleus and can be easily modified by the type, number and position of the substituents. This phenomenon determines their practical application as pigments or dyes in textile, photographic, cosmetic and other industries (Wang *et al.*, 2011). Additionally, they are also known for their anti-inflammatory, wound healing, analgesic, antimicrobial, antitumor and other medicinal properties, which makes them a natural target for pharmaceutical industry (Alves *et al.*, 2004). Due to the favorable structure, anthaquinone derivatives found also numerous applications in supramolecular and electroanalytical chemistry (Czupryniak *et al.*, 2012; Yeh & Wang, 2006). For the above-mentioned reasons, the synthesis of new anthaquinone compounds seems to be important. Here, we present the report on the crystal structure of 1-(piperidin-1-yl)-9,10-anthraquinone.

In the molecule of the title compound (Fig. 1), likewise in the 1-dimethylamino-9,10-anthraquinone (Niedziałkowski *et al.*, 2011) and 1-[methyl(phenyl)amino]anthraquinone (Yatsenko *et al.*, 2000), deviation of planarity of the anthraquinone skeleton is observed. In case of the title compound, such distortion is found to be 0.0885 (3) Å. The piperidine ring adopts a chair conformation, with ring-puckering parameters *Q* = 0.5742 (14) Å, *Θ* = 1.93 (14)° and *φ* = 11 (4)°. The mean planes of piperidine ring and anthracene ring system are inclined at a dihedral angle of 38.7 (1)°. The neighboring anthracene moieties are parallel or inclined at an angle of 63.9 (1)° in the crystal lattice. In the crystal structure, the adjacent molecules are linked by C—H···π (Table 2, Fig. 2) and π–π [centroid-centroid distance = 3.782 (1) Å] (Table 3, Fig. 2) interactions, forming a layer parallel to the *bc* plane.

### Experimental

To 0.5 g 1-chloro-9,10-anthraquinone (2.06 mmol) in 60 ml toluene was added the 177 mg of piperidine (2.05 mmol). The reaction mixture was stirred in 80 °C oil bath for 48 h. After completed reaction, the resulting mixture was evaporated to remove solvent, and dissolved in 150 ml of dichloromethane. The product was washed with water (2 × 100 ml), the organic layer was dried with MgSO_4_, filtered and concentrated. The crude product was purified by silica gel column chromatography using dichloromethane: methanol mixture (5: 0.1). The product was obtained as red solid with yield 90%, 540 mg. Single-crystals were grown by slow evaporation from mixture of methanol and dichloromethane solution at room temperature (m.p. 112–114 °C).

Spectral data: ^1^H NMR (CDCl_3_, 400 MHz): δ (p.p.m.): 1.650–1.711 (p, 2H, –HN–CH_2_–CH_2_–CH_2_–CH_2_–CH_2_–, J_1_ = 4.8 Hz, J_1_ = 5.6 Hz, J_1_ = 6.8 Hz, J_1_ = 7.2 Hz, J_2_ = 5.2 Hz, J_2_ = 5.8 Hz, J_2_ = 6.4 Hz, J_3_ = 11.3 Hz); 1.839–1.895 (p, 4H, –HN–CH_2_–CH_2_–CH_2_–CH_2_–CH_2_–, J_1_ = 5,.2 Hz, J_1_ = 5.6 Hz, J_1_ = 6.0 Hz, J_2_ = 5.4 Hz, J_2_ = 5.6 Hz, J_3_ = 11.0 Hz); 3.187–3,213 (t, 4H, –HN–CH_2_–CH_2_–CH_2_–CH_2_–CH_2_–, J_1_ = 5.2 Hz, J_1_ = 5.6 Hz, J_2_ = 5.4 Hz); 7.390–7.410 (d, 1H, H-2 Ar, Hz, J_2_ = 8.0 Hz); 7.575–7.616 (t, 1H, H-3 Ar, J_1_ = 8.0 Hz, J_1_ = 8.4 Hz, J_2_ = 8.2); 7.698–7.720 (dt, 1H, H-6 Ar, J_1_ = 1.2 Hz, J_1_ = 7.4 Hz, J_1_ = 7.6 Hz, J_2_ = 7.5 Hz); 7.738–7.779 (dt, 1H, H-7 Ar, J_1_ = 0.8 Hz, J_1_ = 1.2 Hz, J_1_ = 7.4 Hz, J_1_ = 7.8 Hz, J_2_ = 7.6 Hz); 7.869–7.889 (dd, 1H, H-4 Ar, J_1_ = 0.8 Hz, J_2_ = 7.2); 8.211–8.233 (dd, 1H, H-5 Ar, J_1_ = 1.2 Hz, J_2_ = 7.6); 8.273–8.295 (dd, 1H, H-8 Ar, J_1_ = 1.2 Hz, J_2_ = 7.2 Hz). IR (KBr): 2943, 2808, 1667, 1648, 1580, 1424, 1312, 1260, 1240, 1132, 1081, 896, 708 cm^-1^. MALDI TOF MS: m/*z* 292.1 [*M*+H]^+^, (MW = 291.34). Elemental analysis: calculated for C_19_H_17_NO_2_: C 78.33, H 5.88, N 4.81; found: C 78.34, H 5.88, N 4.82.

### Refinement

H atoms were positioned geometrically, with C—H = 0.93 Å and 0.97 Å for the aromatic and methylene H atoms, respectively, and constrained to ride on their parent atoms with *U*_iso_(H) = *xU*_eq_(C), where *x* = 1.2 for the aromatic and *x* = 1.5 for the methylene H atoms.

### Crystal data

**Table:**

C_19_H_17_NO_2_	*F*(000) = 616
*M**_r_* = 291.34	*D*_x_ = 1.336 Mg m^−^^3^
Monoclinic, *P*2_1_/*c*	Mo *K*α radiation, λ = 0.71073 Å
Hall symbol: -P 2ybc	Cell parameters from 10180 reflections
*a* = 16.7798 (4) Å	θ = 3.4–29.3°
*b* = 6.84599 (14) Å	µ = 0.09 mm^−^^1^
*c* = 12.6126 (3) Å	*T* = 295 K
β = 90.723 (2)°	Plate, red
*V* = 1448.75 (6) Å^3^	0.42 × 0.35 × 0.05 mm
*Z* = 4	

### Data collection

**Table:**

Oxford Diffraction GEMINI R ULTRA Ruby CCD diffractometer	2565 independent reflections
Radiation source: Enhance (Mo) X-ray Source	2189 reflections with *I* > 2σ(*I*)
Graphite monochromator	*R*_int_ = 0.023
Detector resolution: 10.4002 pixels mm^-1^	θ_max_ = 25.1°, θ_min_ = 3.4°
ω scans	*h* = −20→20
Absorption correction: multi-scan (*CrysAlis RED*; Oxford Diffraction, 2008)	*k* = −8→8
*T*_min_ = 0.969, *T*_max_ = 0.996	*l* = −15→15
18914 measured reflections	

### Refinement

**Table:**

Refinement on *F*^2^	Primary atom site location: structure-invariant direct methods
Least-squares matrix: full	Secondary atom site location: difference Fourier map
*R*[*F*^2^ > 2σ(*F*^2^)] = 0.039	Hydrogen site location: inferred from neighbouring sites
*wR*(*F*^2^) = 0.106	H-atom parameters constrained
*S* = 1.04	*w* = 1/[σ^2^(*F*_o_^2^) + (0.0585*P*)^2^ + 0.233*P*] where *P* = (*F*_o_^2^ + 2*F*_c_^2^)/3
2565 reflections	(Δ/σ)_max_ < 0.001
199 parameters	Δρ_max_ = 0.12 e Å^−^^3^
0 restraints	Δρ_min_ = −0.21 e Å^−^^3^

### Special details

**Table:**

Geometry. All e.s.d.'s (except the e.s.d. in the dihedral angle between two l.s. planes) are estimated using the full covariance matrix. The cell e.s.d.'s are taken into account individually in the estimation of e.s.d.'s in distances, angles and torsion angles; correlations between e.s.d.'s in cell parameters are only used when they are defined by crystal symmetry. An approximate (isotropic) treatment of cell e.s.d.'s is used for estimating e.s.d.'s involving l.s. planes.

### Fractional atomic coordinates and isotropic or equivalent isotropic displacement parameters (Å^2^)

**Table:**

	*x*	*y*	*z*	*U*_iso_*/*U*_eq_	
C1	0.25956 (7)	0.80184 (16)	0.58372 (9)	0.0352 (3)	
C2	0.31025 (8)	0.95054 (19)	0.62053 (11)	0.0451 (3)	
H2	0.2900	1.0455	0.6655	0.054*	
C3	0.38886 (9)	0.9603 (2)	0.59230 (12)	0.0530 (4)	
H3	0.4212	1.0592	0.6195	0.064*	
C4	0.42001 (8)	0.82494 (19)	0.52427 (11)	0.0473 (3)	
H4	0.4729	0.8347	0.5036	0.057*	
C5	0.40087 (8)	0.22450 (19)	0.31080 (10)	0.0454 (3)	
H5	0.4535	0.2399	0.2900	0.054*	
C6	0.35703 (9)	0.0683 (2)	0.27434 (11)	0.0532 (4)	
H6	0.3799	−0.0223	0.2290	0.064*	
C7	0.27893 (10)	0.0461 (2)	0.30517 (12)	0.0574 (4)	
H7	0.2488	−0.0573	0.2785	0.069*	
C8	0.24507 (9)	0.17570 (18)	0.37514 (11)	0.0476 (3)	
H8	0.1928	0.1572	0.3968	0.057*	
C9	0.25025 (7)	0.47080 (18)	0.48949 (10)	0.0397 (3)	
C10	0.41166 (7)	0.53500 (18)	0.41201 (10)	0.0405 (3)	
C11	0.29271 (7)	0.65477 (17)	0.51749 (9)	0.0344 (3)	
C12	0.37301 (7)	0.67421 (17)	0.48642 (9)	0.0369 (3)	
C13	0.28871 (7)	0.33387 (17)	0.41345 (9)	0.0370 (3)	
C14	0.36646 (7)	0.35977 (17)	0.37891 (9)	0.0371 (3)	
N15	0.18002 (6)	0.80008 (15)	0.61441 (8)	0.0378 (3)	
C16	0.11888 (7)	0.8051 (2)	0.53068 (10)	0.0455 (3)	
H16A	0.1361	0.7265	0.4712	0.055*	
H16B	0.1119	0.9383	0.5061	0.055*	
C17	0.04046 (8)	0.7281 (2)	0.57052 (12)	0.0547 (4)	
H17A	0.0463	0.5918	0.5901	0.066*	
H17B	0.0004	0.7369	0.5146	0.066*	
C18	0.01381 (8)	0.8450 (2)	0.66587 (12)	0.0561 (4)	
H18A	0.0013	0.9777	0.6444	0.067*	
H18B	−0.0339	0.7871	0.6950	0.067*	
C19	0.07945 (8)	0.8471 (2)	0.74937 (11)	0.0516 (4)	
H19A	0.0638	0.9307	0.8076	0.062*	
H19B	0.0869	0.7161	0.7770	0.062*	
C20	0.15707 (8)	0.91992 (19)	0.70485 (10)	0.0452 (3)	
H20A	0.1512	1.0549	0.6826	0.054*	
H20B	0.1984	0.9142	0.7592	0.054*	
O21	0.18687 (6)	0.42388 (15)	0.52784 (10)	0.0682 (3)	
O22	0.47785 (6)	0.56781 (15)	0.37776 (9)	0.0607 (3)	

### Atomic displacement parameters (Å^2^)

**Table:**

	*U*^11^	*U*^22^	*U*^33^	*U*^12^	*U*^13^	*U*^23^
C1	0.0364 (6)	0.0372 (6)	0.0320 (6)	−0.0020 (5)	−0.0002 (5)	0.0035 (5)
C2	0.0474 (8)	0.0424 (7)	0.0457 (7)	−0.0052 (5)	0.0029 (6)	−0.0085 (6)
C3	0.0491 (8)	0.0500 (8)	0.0600 (9)	−0.0166 (6)	0.0019 (7)	−0.0124 (7)
C4	0.0375 (7)	0.0500 (8)	0.0546 (8)	−0.0115 (5)	0.0059 (6)	−0.0026 (6)
C5	0.0478 (7)	0.0490 (7)	0.0395 (7)	0.0071 (6)	0.0057 (6)	0.0011 (6)
C6	0.0666 (10)	0.0464 (8)	0.0468 (8)	0.0066 (7)	0.0065 (7)	−0.0079 (6)
C7	0.0716 (10)	0.0429 (8)	0.0577 (9)	−0.0096 (7)	0.0036 (8)	−0.0115 (6)
C8	0.0500 (8)	0.0424 (7)	0.0505 (8)	−0.0081 (6)	0.0053 (6)	−0.0017 (6)
C9	0.0374 (7)	0.0398 (7)	0.0420 (7)	−0.0059 (5)	0.0066 (5)	−0.0001 (5)
C10	0.0382 (7)	0.0450 (7)	0.0386 (6)	−0.0029 (5)	0.0053 (5)	0.0048 (5)
C11	0.0359 (6)	0.0355 (6)	0.0319 (6)	−0.0035 (5)	0.0009 (5)	0.0027 (5)
C12	0.0365 (6)	0.0380 (6)	0.0363 (6)	−0.0043 (5)	0.0030 (5)	0.0041 (5)
C13	0.0422 (7)	0.0338 (6)	0.0350 (6)	−0.0018 (5)	0.0019 (5)	0.0040 (5)
C14	0.0408 (7)	0.0385 (6)	0.0319 (6)	0.0010 (5)	0.0017 (5)	0.0045 (5)
N15	0.0361 (6)	0.0443 (6)	0.0331 (5)	0.0007 (4)	0.0007 (4)	−0.0050 (4)
C16	0.0405 (7)	0.0576 (8)	0.0384 (7)	−0.0002 (6)	−0.0027 (5)	−0.0008 (6)
C17	0.0401 (7)	0.0658 (9)	0.0583 (9)	−0.0045 (6)	−0.0018 (6)	−0.0042 (7)
C18	0.0413 (8)	0.0607 (9)	0.0667 (10)	0.0037 (6)	0.0112 (7)	0.0017 (7)
C19	0.0535 (8)	0.0564 (8)	0.0452 (8)	0.0071 (6)	0.0129 (6)	−0.0030 (6)
C20	0.0480 (7)	0.0464 (7)	0.0414 (7)	0.0033 (6)	0.0025 (6)	−0.0084 (6)
O21	0.0557 (6)	0.0568 (6)	0.0930 (8)	−0.0234 (5)	0.0358 (6)	−0.0244 (6)
O22	0.0451 (6)	0.0679 (7)	0.0696 (7)	−0.0129 (5)	0.0234 (5)	−0.0109 (5)

### Geometric parameters (Å, º)

**Table:**

C1—N15	1.3943 (15)	C10—O22	1.2176 (15)
C1—C2	1.4020 (17)	C10—C14	1.4766 (17)
C1—C11	1.4257 (17)	C10—C12	1.4917 (18)
C2—C3	1.3722 (19)	C11—C12	1.4142 (17)
C2—H2	0.9300	C13—C14	1.3919 (17)
C3—C4	1.3707 (19)	N15—C20	1.4608 (15)
C3—H3	0.9300	N15—C16	1.4634 (16)
C4—C12	1.3804 (17)	C16—C17	1.5094 (19)
C4—H4	0.9300	C16—H16A	0.9700
C5—C6	1.374 (2)	C16—H16B	0.9700
C5—C14	1.3934 (18)	C17—C18	1.517 (2)
C5—H5	0.9300	C17—H17A	0.9700
C6—C7	1.380 (2)	C17—H17B	0.9700
C6—H6	0.9300	C18—C19	1.514 (2)
C7—C8	1.379 (2)	C18—H18A	0.9700
C7—H7	0.9300	C18—H18B	0.9700
C8—C13	1.3905 (18)	C19—C20	1.5094 (19)
C8—H8	0.9300	C19—H19A	0.9700
C9—O21	1.2171 (15)	C19—H19B	0.9700
C9—C11	1.4873 (17)	C20—H20A	0.9700
C9—C13	1.4936 (17)	C20—H20B	0.9700
			
N15—C1—C2	119.55 (11)	C14—C13—C9	122.36 (11)
N15—C1—C11	122.58 (10)	C13—C14—C5	120.37 (12)
C2—C1—C11	117.86 (11)	C13—C14—C10	119.68 (11)
C3—C2—C1	122.03 (12)	C5—C14—C10	119.92 (11)
C3—C2—H2	119.0	C1—N15—C20	118.34 (10)
C1—C2—H2	119.0	C1—N15—C16	117.66 (9)
C4—C3—C2	120.34 (12)	C20—N15—C16	111.16 (10)
C4—C3—H3	119.8	N15—C16—C17	110.97 (11)
C2—C3—H3	119.8	N15—C16—H16A	109.4
C3—C4—C12	120.06 (12)	C17—C16—H16A	109.4
C3—C4—H4	120.0	N15—C16—H16B	109.4
C12—C4—H4	120.0	C17—C16—H16B	109.4
C6—C5—C14	119.96 (13)	H16A—C16—H16B	108.0
C6—C5—H5	120.0	C16—C17—C18	110.29 (12)
C14—C5—H5	120.0	C16—C17—H17A	109.6
C5—C6—C7	119.84 (12)	C18—C17—H17A	109.6
C5—C6—H6	120.1	C16—C17—H17B	109.6
C7—C6—H6	120.1	C18—C17—H17B	109.6
C8—C7—C6	120.67 (13)	H17A—C17—H17B	108.1
C8—C7—H7	119.7	C19—C18—C17	109.72 (11)
C6—C7—H7	119.7	C19—C18—H18A	109.7
C7—C8—C13	120.26 (13)	C17—C18—H18A	109.7
C7—C8—H8	119.9	C19—C18—H18B	109.7
C13—C8—H8	119.9	C17—C18—H18B	109.7
O21—C9—C11	123.20 (11)	H18A—C18—H18B	108.2
O21—C9—C13	118.45 (11)	C20—C19—C18	111.59 (11)
C11—C9—C13	118.32 (10)	C20—C19—H19A	109.3
O22—C10—C14	121.14 (12)	C18—C19—H19A	109.3
O22—C10—C12	120.74 (11)	C20—C19—H19B	109.3
C14—C10—C12	118.10 (10)	C18—C19—H19B	109.3
C12—C11—C1	118.43 (10)	H19A—C19—H19B	108.0
C12—C11—C9	117.97 (10)	N15—C20—C19	110.02 (11)
C1—C11—C9	123.22 (11)	N15—C20—H20A	109.7
C4—C12—C11	121.11 (11)	C19—C20—H20A	109.7
C4—C12—C10	116.37 (11)	N15—C20—H20B	109.7
C11—C12—C10	122.52 (11)	C19—C20—H20B	109.7
C8—C13—C14	118.82 (11)	H20A—C20—H20B	108.2
C8—C13—C9	118.81 (11)		
			
N15—C1—C2—C3	−179.53 (12)	O21—C9—C13—C8	11.05 (19)
C11—C1—C2—C3	1.84 (19)	C11—C9—C13—C8	−171.04 (11)
C1—C2—C3—C4	1.5 (2)	O21—C9—C13—C14	−169.48 (12)
C2—C3—C4—C12	−2.1 (2)	C11—C9—C13—C14	8.43 (17)
C14—C5—C6—C7	0.1 (2)	C8—C13—C14—C5	−2.79 (18)
C5—C6—C7—C8	−2.2 (2)	C9—C13—C14—C5	177.74 (11)
C6—C7—C8—C13	1.7 (2)	C8—C13—C14—C10	175.23 (11)
N15—C1—C11—C12	176.97 (10)	C9—C13—C14—C10	−4.24 (17)
C2—C1—C11—C12	−4.45 (16)	C6—C5—C14—C13	2.36 (18)
N15—C1—C11—C9	−10.26 (17)	C6—C5—C14—C10	−175.66 (12)
C2—C1—C11—C9	168.32 (11)	O22—C10—C14—C13	−175.33 (12)
O21—C9—C11—C12	166.47 (13)	C12—C10—C14—C13	2.97 (17)
C13—C9—C11—C12	−11.33 (16)	O22—C10—C14—C5	2.71 (18)
O21—C9—C11—C1	−6.3 (2)	C12—C10—C14—C5	−179.00 (10)
C13—C9—C11—C1	175.87 (10)	C2—C1—N15—C20	−16.08 (16)
C3—C4—C12—C11	−0.7 (2)	C11—C1—N15—C20	162.48 (11)
C3—C4—C12—C10	179.43 (12)	C2—C1—N15—C16	122.15 (12)
C1—C11—C12—C4	3.98 (17)	C11—C1—N15—C16	−59.30 (15)
C9—C11—C12—C4	−169.18 (11)	C1—N15—C16—C17	158.76 (11)
C1—C11—C12—C10	−176.15 (10)	C20—N15—C16—C17	−60.21 (14)
C9—C11—C12—C10	10.69 (17)	N15—C16—C17—C18	57.32 (16)
O22—C10—C12—C4	−8.32 (18)	C16—C17—C18—C19	−54.05 (16)
C14—C10—C12—C4	173.38 (11)	C17—C18—C19—C20	54.41 (16)
O22—C10—C12—C11	171.81 (12)	C1—N15—C20—C19	−159.99 (11)
C14—C10—C12—C11	−6.49 (17)	C16—N15—C20—C19	59.26 (13)
C7—C8—C13—C14	0.77 (19)	C18—C19—C20—N15	−56.84 (15)
C7—C8—C13—C9	−179.74 (12)		

### Hydrogen-bond geometry (Å, º)

*Cg*3 is the centroid of the C5–C8/C13/C14 ring.

**Table:**

*D*—H···*A*	*D*—H	H···*A*	*D*···*A*	*D*—H···*A*
C2—H2···*Cg*3^i^	0.93	2.88	3.685 (2)	146

Symmetry code: (i) *x*, −*y*+1/2, *z*−1/2.

### π–π interaction geometry (Å,°).

**Table:**

*I*	*J*	*CgI*···*CgJ*	Dihedral angle	*CgI*_Perp	*CgJ*_Perp	*CgI*_Offset	*CgJ*_Offset
2	3^ii^	3.782 (1)	3.31 (6)	3.615 (1)	3.660 (1)	1.112 (1)	0.953 (1)

Symmetry code: (ii) *x*, *y* + 1, *z*.Notes: *Cg*2 and *Cg*3 are the centroids of the C1–C4/C11/C12 and C5–C8/C13/C14 rings, respectively. Cg*I*···Cg*J* is the distance between ring centroids. The dihedral angle is that between the planes of the rings *I* and *J*. *CgI*_Perp is the perpendicular distance of *CgI* from ring *J*. *CgJ*_Perp is the perpendicular distance of *CgJ* from ring *I*. Cg*I*_Offset is the distance between *CgI* and perpendicular projection of *CgJ* on ring *I*. Cg*J*_Offset is the distance between *CgJ* and perpendicular projection of *CgI* on ring *J*.
